# Distribution of SET/I2PP2A protein in gastrointestinal tissues

**DOI:** 10.1371/journal.pone.0222845

**Published:** 2019-09-26

**Authors:** Koji Umata, Yusuke Sakai, Shunta Ikeda, Shunya Tsuji, Hideyoshi Kawasaki, Takashi Ohama, Koichi Sato

**Affiliations:** 1 Laboratory of Veterinary Pharmacology, Joint Faculty of Veterinary Medicine, Yamaguchi University, Yamaguchi, Japan; 2 Laboratory of Veterinary Pathology, Joint Faculty of Veterinary Medicine, Yamaguchi University, Yamaguchi, Japan; Istituto Superiore di Sanità, ITALY

## Abstract

SET (also called I2PP2A and TIF-1) is a multi-functional protein that regulates a variety of cell signaling including nucleosome assembly, histone binding, and tumorigenesis. Elevated SET protein levels are observed in various human tumors, and are correlated with poor prognosis and drug-resistance. We recently reported that SET protein levels in cancer cells were positively correlated with poor prognosis of gastric cancer patients. Using immunohistochemistry, SET protein was observed not only in cancer cells, but also in some interstitial cells. However, the tissue distribution of SET has not been investigated. Here we performed co-immunofluorescent staining to characterize SET protein distribution in gastrointestinal tissues. We found that even though the positive rate is much lower than epithelial cells, SET protein is also expressed in non-epithelial cells, such as monocytes/macrophages, neural cells, myofibroblasts, and smooth muscle cells. Our results indicate an extensive role of SET in a variety of cell types.

## Introduction

SET (suvar3-9 enhancer-of-zeste trithorax) is a multi-functional protein that is also called as I2PP2A or TIF-I (template activating factor I). In 1992, the human *SET* gene was discovered as a component of a *SET-CAN* fusion gene in a case of acute undifferentiated leukemia [1]. In 1993, TIF-I protein was isolated from HeLa cell extracts as a protein stimulating adenovirus core DNA replication [2], and in 1995, I2PP2A was purified from bovine brain extract as a heat-stable protein inhibitor of Ser/Thr protein phosphatase 2A (PP2A) [3]. Thereafter, it was independently discovered that TIF-I, I2PP2A and SET are identical [4,5]. There are four highly similar transcriptional variants of human SET that differ in their N-terminal amino acids. Among them, SETα (I2βPP2A or TIF-Iα) and SETβ (I2αPP2A or TIF-Iβ) are major variants and well studied. Both SETα and SETβ exhibit PP2A inhibitory activity [6], however only SETβ has chromatin remodeling activity [7].

Accumulating evidence has revealed the multi-functional roles of SET, including nucleosome assembly, histone binding, transcription control, and cell death [8–10]. Elevated SET protein levels are observed in various human tumors, including colorectal cancer, gastric cancer, pancreatic cancer, breast cancer, non-small cell lung cancer, and acute myeloid leukemia, and SET levels are correlated with poor prognosis and drug-resistance [11–16]. Recently, we found that SET regulates gastric cancer cell stemness by stabilizing transcriptional factor E2F1 protein by suppressing PP2A activity [15]. Immunohistochemistry revealed that SET protein levels in cancer cells were positively correlated with poor prognosis of gastric cancer patients [15]. In that study, we observed that SET protein level is increased not only in cancer cells, but also in some interstitial cells in tumor micro-environment. Therefore, SET protein may play a role in interstitial cells to promote/suppress tumor progression. However, to data, the tissue distribution of SET protein has not been investigated. The gastrointestinal tract consists of various types of cells and useful to analyze the expression pattern of proteins in these different cell types. Here we performed co-immunofluorescence staining to characterize SET protein distribution in gastrointestinal tissues.

## Material and methods

### Mice

C57BL/6J mice purchased from Charles River Japan (Yokohama, Japan) were maintained in compliance with the guidelines of the Animal Care and Use Committee of Yamaguchi University. C57BL/6J mice were anesthetized with diethyl ether and euthanized by exsanguination. All experiments and animal care procedures in this study were performed according to the Guide to Animal Use and Care of the Yamaguchi University and were approved by the ethics committee. Mice were sacrificed by blood removal under isoflurane anesthesia.

### Immunohistochemistry and immunofluorescence staining

Mouse intestinal tissues were fixed in 10% neutral buffered formalin and embedded in paraffin. 4 μm thick sections were cut from tissue blocks, mounted on silane-coated slides, and subsequently de-waxed and rehydrated using xylene and graded alcohol washes.

For immunohistochemistry, antigen retrieval was carried out by autoclaving (121°C, 5 min) in Tris-EDTA buffer solution (pH 9.0). After washing with phosphate buffered saline (PBS), endogenous peroxidase was inactivated by immersion in 3% hydrogen peroxide in PBS. Sections were blocked with PBS containing 10% skim milk/1% bovine serum albumin (BSA) for 30 min, followed by the addition of primary antibodies overnight at 4°C. After incubation with primary antibodies, slides were washed in two changes of PBS and incubated with EnVision+ system-HRP-labeled polymer anti-rabbit (Agilent, CA, USA) or ImmPRESS-HRP labeled anti-Goat IgG (Vector Laboratories Inc. CA, USA). Positive signals were then visualized by peroxidase-diaminobenzidine reaction, and sections were counterstained with hematoxylin.

For immunofluorescence staining, antigen retrieval was carried out by autoclaving (121°C, 5 min) in citrate buffer solution (pH 6.0). Sections were blocked with PBS containing 10% skim milk/1% BSA for 30 min, followed by the addition of primary antibodies overnight at 4°C. After incubation with primary antibodies, slides were washed in two changes of PBS before being incubated with secondary antibodies and Hoechst 33342 (Dojindo, Tokyo, Japan) for 1 h at room temperature. The dilution of antibodies is described in Table 1. Fluorescent images were captured using an HS All-in-One Fluorescent Microscope (BZ-9000, Keyence, Osaka, Japan). At least 3 pictures were randomly taken and more than 100 cells were counted for each mouse. Three mice were used to analyze SET positive rate. The representative pictures of hematoxylin-eosin staining of adjacent regions were shown in S1 Fig.

**Table 1:** List of antibodies used in this study.

Primary Antibodies			
Antigen	Manufacturer	Catalogue Number	Dilution
α-smooth muscle actin	Sigma Aldrich	A-2547	1:200
E-cadherin	BD Biosciences	610181	1:200
Iba1	FUJIFILM Wako	019–19741	1:200
Ki67	Agilent	M7249	1:200
PGP9.5	Agilent	Z511601-2	1:200
SET	Santa Cruz	sc-5655	1:400
SET	Bioss	bs-5943	1:50
Secondary Antibodies			
Anti-Mouse IgG, Alexa Fluor 594	ThermoFisher	A-21203	1:1000
Anti-Rat IgG, Alexa Fluor 594	ThermoFisher	A-21209	1:1000
Anti-Rabbit IgG, Alexa Fluor 594	ThermoFisher	A-21207	1:1000
Anti-Goat IgG, Alexa Fluor 488	ThermoFisher	A-11055	1:1000

### Immunoblotting

Immunoblotting was performed as previously described [15]. Tissue samples were lysed in a buffer containing 50 mM Tris-HCl (pH 8.0), 5 mM EDTA, 5 mM EGTA, 1% Triton X100, 1 mM Na_3_VO_4_, 20 mM sodium pyrophosphate, and Roche Complete protease inhibitor mixture. Proteins were separated by SDS-PAGE and transferred onto nitrocellulose membrane (Fujifilm, Osaka, Japan). The membranes were blocked with 0.5% skim milk and treated with primary antibodies, and immunoreactive bands were visualized using ECL Pro (PerkinElmer, MA, USA) and LAS-3000 (Fujifilm). Band densities were quantified using ImageJ densitometry analysis software (National Institutes of Health). p97/VCP (Valosin Containing Protein) was used as a loading control.

### Statistical analysis

Statistical analysis was performed using SigmaPlot (HULINKS, Tokyo, Japan). The results are expressed as mean ± S.E. Groups were compared using one-way analysis of variance (Degree of freedom of between groups is 4), after which the Fisher LSD test was used. For all analyses, a probability value of *p* < 0.05 was considered statistically significant.

## Results

### Distribution of SET protein in mouse gastrointestinal tissues

To qualify the specificity of SET antibodies, we first performed immunohistochemistry with two different anti-SET antibodies (SantaCruz sc-5655 and Bioss bs-5943). Similar staining pattern was observed by two antibodies (S2 Fig). As we previously observed in human gastric tissues, SET protein was observed not only in epithelial cells, but also in some interstitial cells. Therefore, we analyzed tissue distribution of SET by immunofluorescence double staining. Because Bioss’s antibody showed relatively higher background, we utilized SantaCruz’s anti-SET antibody for immunofluorescence. This antibody reacts with both SETα and SETβ. Immunoblotting showed similar band pattern throughout gastrointestinal tract (S3 Fig).

### About 80% of Ki67-positive cells are SET-positive

**Fig 1:**
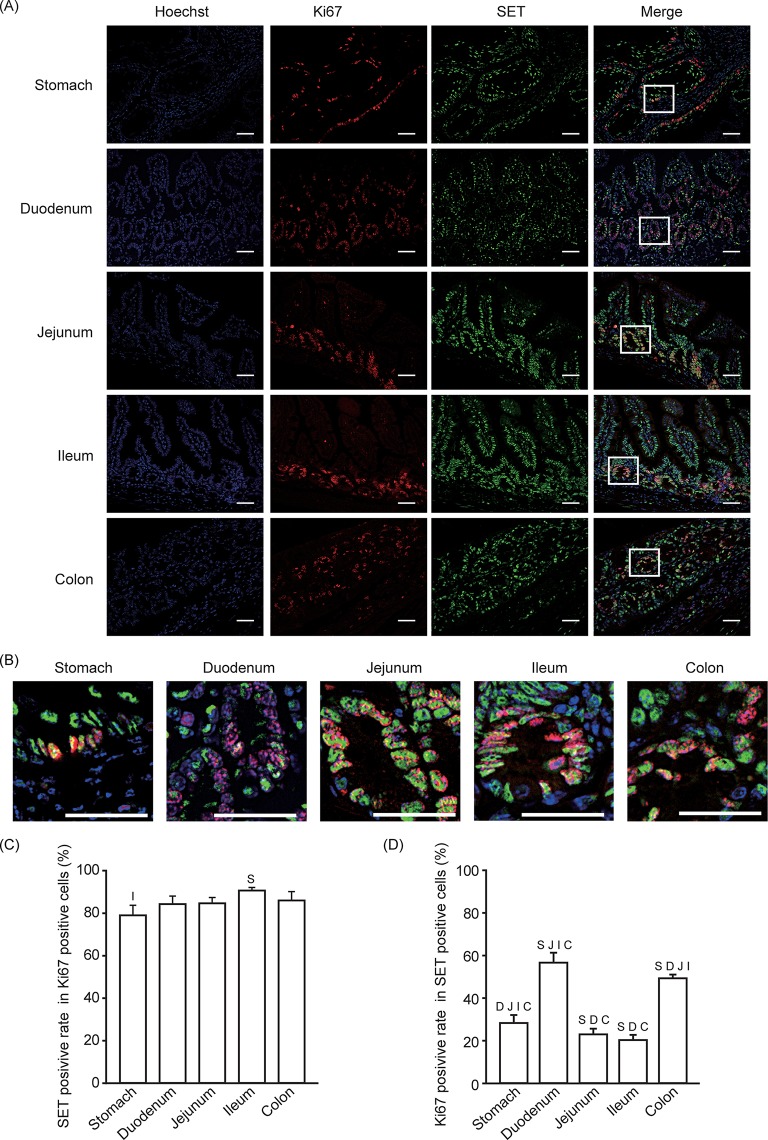
About 80% of the Ki67-positive cells are SET-positive. Immunofluorescence staining was performed to analyze SET and Ki67 expression in gastrointestinal tissues from mice. (A, B) Representative pictures of SET (Green), Ki67 (Red), Hoechist 33342 (Blue), and merge are shown. Enlarged pictures of indicated area in (A) are shown in (B). Scale bars: 40 μm. (C, D) Quantitative data (means ± S.E.) from 3 mice. (C) SET positive rate in Ki67 positive cells and (D) Ki67 positive rate in SET positive cells are shown. S: *p* < 0.05 vs. Stomach, D: *p* < 0.05 vs. Duodenum, J: *p* < 0.05 vs. Jejunum, I: *p* < 0.05 vs. Ileum, C: *p* < 0.05 vs. Colon.

The suppression of SET expression leads to decreased cell proliferation in various types of cancer cells [13,17–19]. Therefore, we analyzed the co-localization of SET and the proliferation marker Ki67 by co-immunofluorescent staining (Fig 1A and 1B). As we and others have previously described, SET abundantly localized in the nucleus [17,20]. We found that about 80% of Ki67-positive cells are also positive for SET throughout the gastrointestinal tract (Fig 1C). On the other hand, the ratio of Ki67-positive in SET-positive cells depends on the area: from ~20–60% (Fig 1D), indicating that not all SET-positive cells are in a proliferation state.

### SET-positive rate in epithelial cells

**Fig 2:**
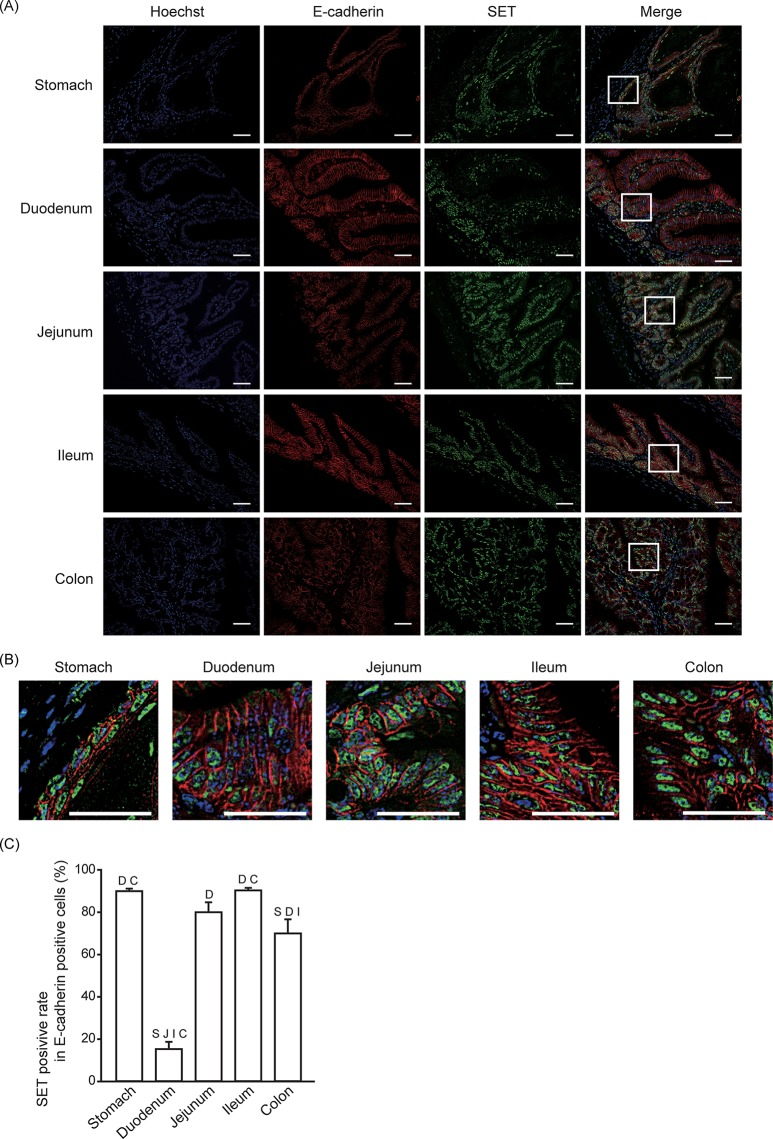
SET-positive rate in epithelial cells. Immunofluorescence staining was performed to analyze SET and E-cadherin expression in gastrointestinal tissues from mice. (A, B) Representative pictures of SET (Green), E-cadherin (Red), Hoechist 33342 (Blue), and merge are shown. Enlarged pictures of indicated area in (A) are shown in (B). Scale bars: 40 μm. (C) Quantitative data (means ± S.E.) for SET positive rate in E-cadherin positive cells are shown. N = 3. S: *p* < 0.05 vs. Stomach, D: *p* < 0.05 vs. Duodenum, J: *p* < 0.05 vs. Jejunum, I: *p* < 0.05 vs. Ileum, C: *p* < 0.05 vs. Colon.

Increased SET expression is correlated with poor prognosis in patients with gastric cancer and metastatic colorectal cancer [12,17]. We previously reported that SET is expressed in human gastric epithelial cells although it is much weaker than in gastric cancer cells [17]. Therefore, we analyzed SET protein expression in epithelial cells by using E-cadherin as an epithelial marker (Fig 2A and 2B). In most areas of the gastrointestinal tract, a high percentage (about 80%) of epithelial cells were SET positive (Fig 2C). Interestingly, in the upper part of the small intestine (duodenum), SET was mainly expressed in the bottom of the villi and the SET-positive rate in E-cadherin-positive cells was very low (less than 20%).

### SET-positive rate in monocytes/macrophages

**Fig 3:**
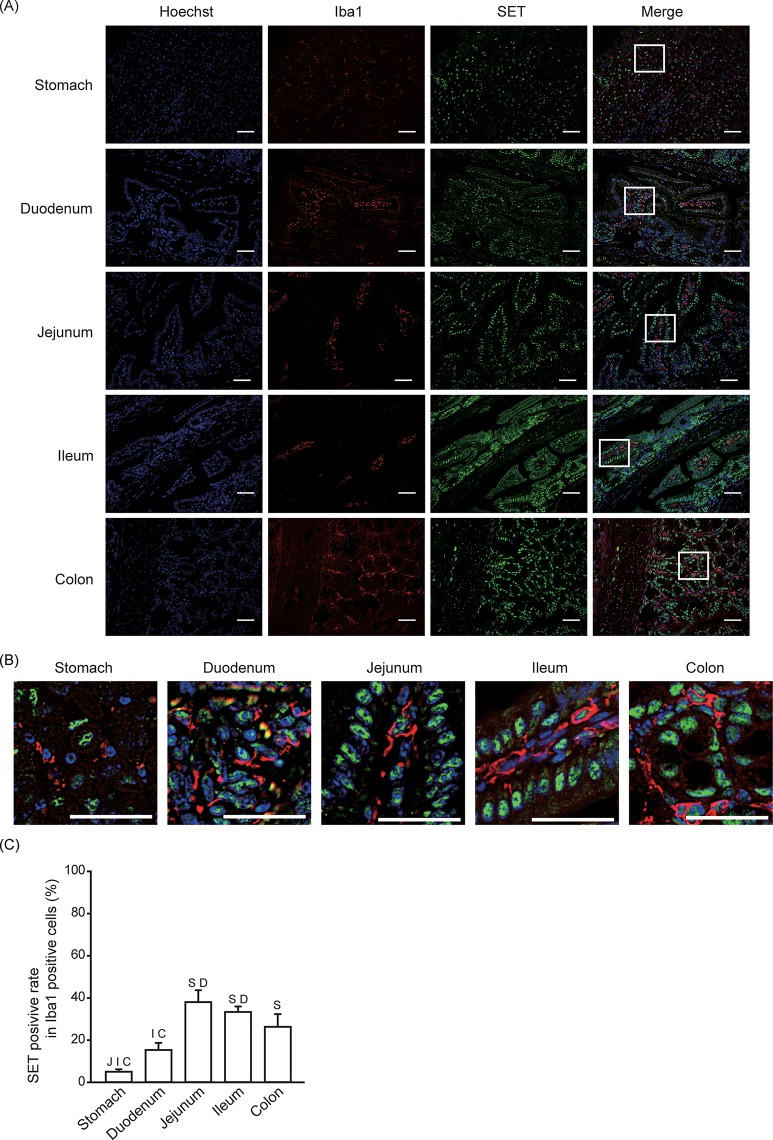
SET-positive rate in monocytes/macrophages. Immunofluorescence staining was performed to analyze SET and Iba1 expression in gastrointestinal tissues from mice. (A, B) Representative pictures of SET (Green), Iba1 (Red), Hoechist 33342 (Blue), and merge are shown. Enlarged pictures of indicated area in (A) are shown in (B). Scale bars: 40 μm. (C) Quantitative data (means ± S.E.) for SET positive rate in Iba1 positive cells are shown. N = 3. S: *p* < 0.05 vs. Stomach, D: *p* < 0.05 vs. Duodenum, J: *p* < 0.05 vs. Jejunum, I: *p* < 0.05 vs. Ileum, C: *p* < 0.05 vs. Colon.

Given that we observed SET expression in the submucosa and smooth muscle layer, we analyzed whether hematopoietic cells express SET. We stained for Iba1 as a marker for monocytes/macrophages (Fig 3A and 3B). Iba1-positive cells are mostly localized in the subepithelial area. The SET-positive rate in monocytes/macrophages was relatively low (less than 40%), and tended to lower in the upper part of the gastrointestinal tract (Fig 3C).

### SET-positive rate in neural cells

**Fig 4:**
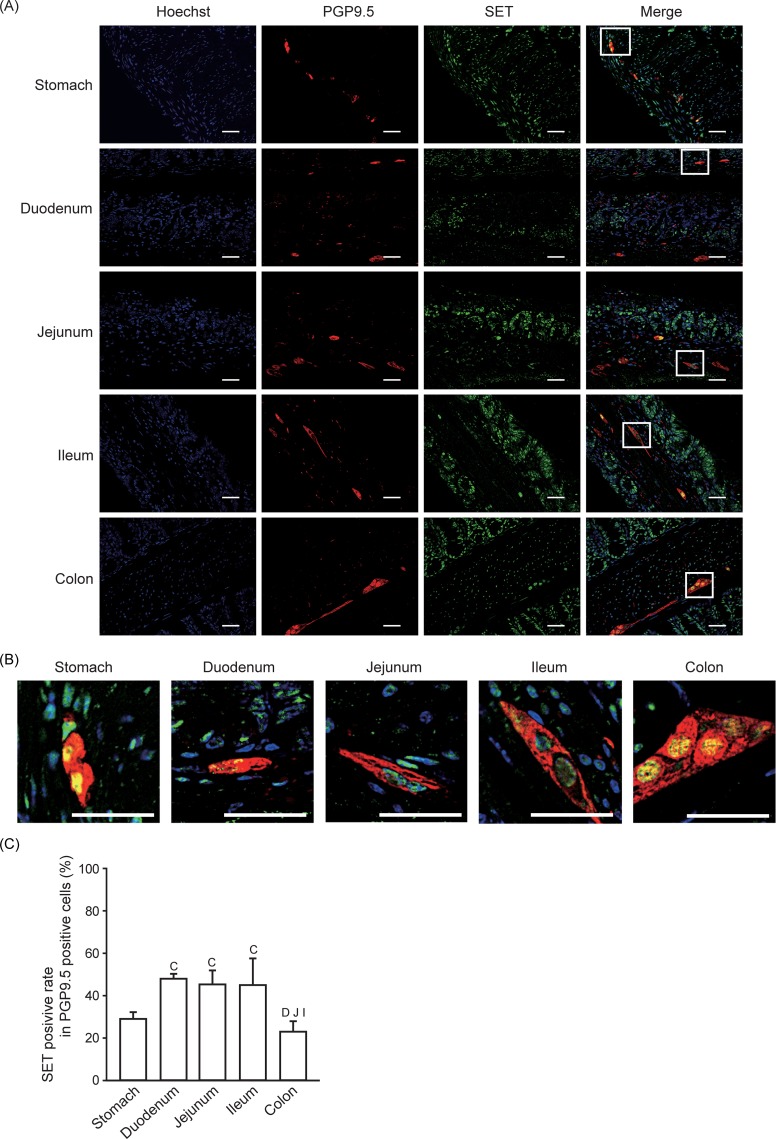
SET-positive rate in neural cells. Immunofluorescence staining was performed to analyze SET and PGP9.5 expression in gastrointestinal tissues from mice. (A, B) Representative pictures of SET (Green), PGP9.5 (Red), Hoechist 33342 (Blue), and merge are shown. Enlarged pictures of indicated area in (A) are shown in (B). Scale bars: 40 μm. (C) Quantitative data (means ± S.E.) for SET positive rate in PGP9.5 positive cells are shown. N = 3. D: *p* < 0.05 vs. Duodenum, J: *p* < 0.05 vs. Jejunum, I: *p* < 0.05 vs. Ileum, C: *p* < 0.05 vs. Colon.

Next, we analyzed SET expression in neural cells by using PGP9.5 as a marker (Fig 4A and 4B). Anti-PGP9.5 antibody labels the neuronal cell bodies and axons. Given that SET is predominantly expressed in nucleus, we analyzed the percentage of SET-positive nuclei per total number of neural nuclei (cell body). We found that less than 50% of neural cells express SET (Fig 4C).

### SET-positive rate in myofibroblasts and smooth muscle cells

**Fig 5:**
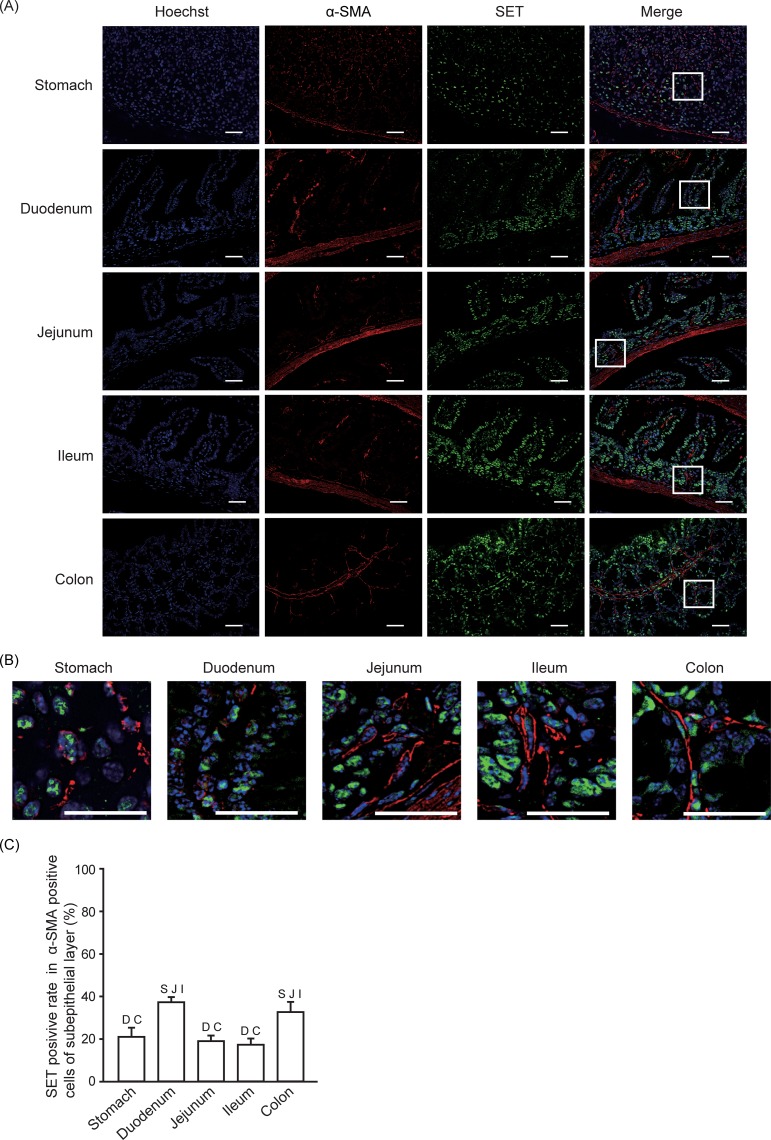
SET-positive rate in myofibroblasts. Immunofluorescence staining was performed to analyze SET and α-SMA expression in gastrointestinal tissues from mice. (A) Representative pictures of SET (Green), α-SMA (Red), Hoechist 33342 (Blue), and merge are shown. Enlarged pictures of indicated area in (A) are shown in (B). Scale bars: 40 μm. (C) Quantitative data (means ± S.E.) for SET positive rate in α-SMA positive cells in subepithelial layer are shown. N = 3. S: *p* < 0.05 vs. Stomach, D: *p* < 0.05 vs. Duodenum, J: *p* < 0.05 vs. Jejunum, I: *p* < 0.05 vs. Ileum, C: *p* < 0.05 vs. Colon.

**Fig 6:**
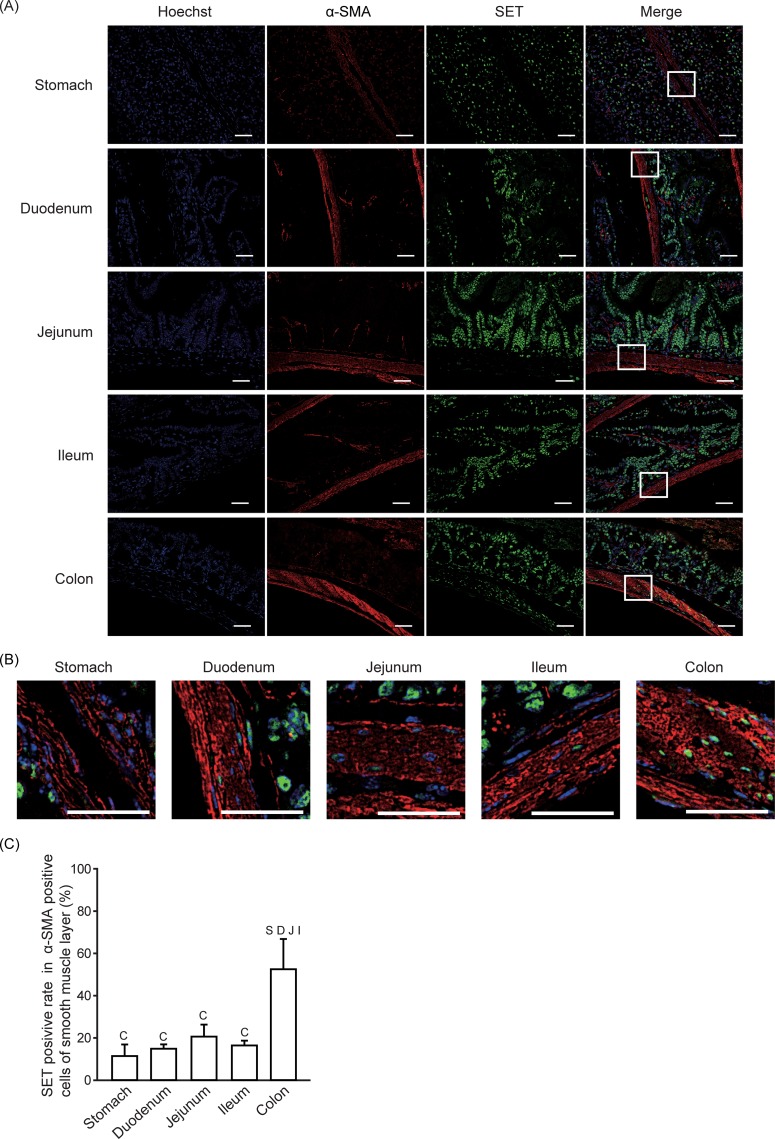
SET-positive rate in smooth muscle cells. Immunofluorescence staining was performed to analyze SET and α-SMA expression in gastrointestinal tissues from mice. (A) Representative pictures of SET (Green), α-SMA (Red), Hoechist 33342 (Blue), and merge are shown. Enlarged pictures of indicated area in (A) are shown in (B). Scale bars: 40 μm. (C) Quantitative data (means ± S.E.) for SET positive rate in α-SMA positive cells in smooth muscle layer are shown. N = 3. S: *p* < 0.05 vs. Stomach, D: *p* < 0.05 vs. Duodenum, J: *p* < 0.05 vs. Jejunum, I: *p* < 0.05 vs. Ileum, C: *p* < 0.05 vs. Colon.

Finally, we analyzed SET expression in subepithelial myofibroblasts and smooth muscle cells (Fig 5A and 5B and Fig 6A and 6B). Myofibroblasts are identified as cells located close to the basal surface of epithelial cells. We found that 20–40% of subepithelial myofibroblasts express SET (Fig 5C). In smooth muscle cells, the SET-positive rate was very low from the stomach and the small intestine, while it was exceptionally high (about 60%) in the colon (Fig 6C).

## Discussion

In this report, we analyzed the distribution of SET protein in gastrointestinal tissues. Elevated SET protein levels are observed in various human tumors, and are positively correlated with the poor prognosis of patients with several types of cancer [11,15,16]. Therefore, it is quite important to know what types of cells express SET protein in normal tissues. The gastrointestinal tract consists of various types of cells, which makes it essential to investigate the SET expression pattern in each cell type individually. Our observation revealed that various types of cells express SET, however the positive rate for SET protein varies substantially between cell types and the regions of the gastrointestinal tract.

Previous reports indicate that SET positively regulates cell proliferation [13,17–19]. Consistent with this, a high percentage of proliferating cells observed in this study expressed SET (Fig 1C). However, the reverse was not always true in that the Ki67-positive rate in SET-positive cells was low (e.g. about 20% in ileum) (Fig 1D). Most of the Ki67-positive cells are located in the epithelial layer, especially the area where stem/progenitor cells are located, such as the bottom of the crypt in the small intestine. In duodenum, SET is mainly observed in the crypt, but not in the villi, leading to the low SET-positive rate in E-cadherin-positive cells (Fig 2C), but the relatively high SET-positive rate in Ki67 positive cells (Fig 1C). Low SET-positive rate in differentiated epithelial cells in duodenum may cause lower band density of SET in immunoblotting (S3 Fig). SET expression in the stem/progenitor cells is consistent with the role of SET in the maintenance of cell stemness [15]. However, in the other regions of the gastrointestinal tract, SET is also expressed in the differentiated epithelial cells, leading to a high SET-positive rate in E-cadherin-positive cells (Fig 2C). These data suggest that SET expression may fill the necessary conditions but not be sufficient for the maintenance of cell stemness.

Although the positive rate is much lower than epithelial cells, SET is also expressed in non-epithelial cells, such as monocytes/macrophages, neural cells, myofibroblasts, and smooth muscle cells. Moreover, SET expression was observed in the lymphocyte lineage, such as acute/chronic leukemia cells and natural killer cells [16,21,22]. To our knowledge, this is the first report showing that monocytes/macrophages express SET protein (Fig 3). The role of SET in these cells remains unknown, but the relatively low positive rate suggests that only a subset of monocytes/macrophages express SET. Accumulating evidence suggests that SET may play a neuroprotective roles in the central nervous system [23–26]. Taken together with our data (Fig 4), SET may also play a protective role in peripheral neural cells. Interestingly, only about 5% of smooth muscle cells within the stomach and small intestine express SET protein, while the SET-positive rate of colonic smooth muscle cells is more than 60% (Fig 5). Currently, there is no report examining the role of SET in smooth muscle cells, so further investigation is necessary to clarify the reason behind this substantial difference.

There are four highly similar transcriptional variants of human SET that differ in their N-terminal amino acids. Among them, SETα and SETβ are highly expressed and well studied. Several studies have revealed the multifunctional roles of SET, but the functional difference between these two isoforms is not completely clear. It was reported that both isoforms inhibit PP2A activity [6], however only SETβ has chromatin remodeling activity [7]. The antibody used in this study cannot distinguish the isoforms in immunostaining, and at the moment, the antibody specific for SETα is not available. Given that it has been reported that the SETα/ SETβ protein ratio correlates with parameters of chronic lymphocytic leukemia [18], the generation of a SETα-specific antibody is required.

## Supporting information

S1 Fig Hematoxylin-eosin staining of adjacent regions. Hematoxylin-eosin staining was performed to show the morphological feature of indicated area used for immunostaining. Scale bars: 50 μm.
(EPS)

S2 Fig Immunohistochemistry with anti-SET antibodies. Immunohistochemistry was performed with anti-SET antibodies from SantaCruz (A) and Bioss (B). Scale bars: 100 μm.
(EPS)

S3 Fig Immunoblotting with anti-SET antibodies. Mouse gastrointestinal tissues were lysed in a buffer as mentioned in Material and Methods, and the extracts were applied for immunoblotting with anti-SET antibodies from SantaCruz. VCP was used as a loading control.
(EPS)
